# Superior hydrogen storage in high entropy alloys

**DOI:** 10.1038/srep36770

**Published:** 2016-11-10

**Authors:** Martin Sahlberg, Dennis Karlsson, Claudia Zlotea, Ulf Jansson

**Affiliations:** 1 Department of Chemistry - Ångström Laboratory, Uppsala University, Box 523, SE-75120 Uppsala, Sweden; 2 Université Paris Est, Institut de Chimie et des Matériaux de Paris-Est, (UMR7182), CNRS-UPEC, 2-8 rue Henri Dunant, F-94320 Thiais, France

## Abstract

Metal hydrides (MH_x_) provide a promising solution for the requirement to store large amounts of hydrogen in a future hydrogen-based energy system. This requires the design of alloys which allow for a very high H/M ratio. Transition metal hydrides typically have a maximum H/M ratio of 2 and higher ratios can only be obtained in alloys based on rare-earth elements. In this study we demonstrate, for the first time to the best of our knowledge, that a high entropy alloy of TiVZrNbHf can absorb much higher amounts of hydrogen than its constituents and reach an H/M ratio of 2.5. We propose that the large hydrogen-storage capacity is due to the lattice strain in the alloy that makes it favourable to absorb hydrogen in both tetrahedral and octahedral interstitial sites. This observation suggests that high entropy alloys have future potential for use as hydrogen storage materials.

Hydrogen has a strong potential for use as an alternative fuel provided that it can be stored in a safe and efficient way. One possibility is to store hydrogen as a solid hydride using suitable metals or alloys. Metal hydrides have been widely studied as storage materials but most alloys are unable to fulfil the requirements of a competitive hydrogen storage unit that can be exploited in practical applications. A problem with hydrides based only on transition metals is their limited capacity with an H/M ratio ≤ 2. Metal hydrides used for applications today (e.g. AB_5_-type) have acceptable storage capability but require the use of rare-earth metals such as lanthanum. Consequently, there is a need for new concepts to identify more efficient hydrogen storage alloys^1^. In this letter we will demonstrate such a design concept based on high entropy alloys (HEA).

It is well-known that metals and alloys with a BCC-type structure have a large capacity to store hydrogen (see e.g. refs 1, 2, 3). It is also known that the presence of lattice strain in the lattice or at interfaces in some metals can be favourable for hydride formation (see e.g. ref. 4). These two factors suggest that high entropy alloys (HEA) can have excellent hydrogen storage potential due to their important lattice strain. The concept of HEAs was originally demonstrated by Yeh *et al*.^5^ and Cantor *et al*.^6^ in 2004. In an HEA, five or more elements are mixed in approximately equimolar ratios. HEAs typically crystallize in a simple structure, often BCC or FCC (cubic close packing). The formation of a multicomponent solid solution is favoured by the high entropy of mixing. A typical feature of an HEA is that the lattice is distorted due to the variation in the atomic radii of the constituent atoms. This distortion will lead to a strained lattice which may be beneficial for hydride formation. Hitherto, hydrogen storage in HEAs has been reported in a few papers with limited success. For example, Kunce *et al*.^7^ investigated the properties of ZrTiVCrFeNi, where they observed storage of 1.81 wt% hydrogen at 100 bar and 50 °C after activation at 500 °C after synthesis. After further heat treatments the maximum hydrogen content decreased to 1.56 wt% at the same hydrogenation conditions. They also studied TiZrNbMoV^8^ and found lower hydrogen storage (0.59 wt%) in the single phase BCC-type structure as compared to the multiphase material (2.3 wt%), at a hydrogen pressure of 85 bar at 50 °C.

A more efficient hydride-forming HEA could be TiZrHfNbV that is known to crystallize in a BCC-type structure. The alloy contains elements which are strong hydride formers. Moreover, the large difference in atomic radii of the elements (δ = 6.82%) suggest a significant lattice distortion that could favour a more efficient hydrogen storage capacity. The aim of the present work has been to investigate hydride formation in TiZrHfNbV and to evaluate its potential for use as a hydrogen storage material.

## Methods

Equimolar TiVZrNbHf samples were synthesized by arc-melting stoichiometric amounts of Ti (99.995% pure, Chempur), V (99.95% pure, MRC), Zr (99.8% pure, Chempur). Nb (99.8% pure, Cerac) and Hf (99.6% pure, Johnson Matthey, Materials and Technology U.K.). To ensure homogeneity, the sample was re-melted five times and turned over between each melting step. Oxygen contamination was minimized by flushing the furnace three times with high purity Ar and by melting a Ti getter for 5 minutes prior to each cycle of alloy melting. The total weight loss from the sample was less than 1%. The chemical composition and microstructure was studied using a Zeiss 1550 scanning electron microscope (SEM) equipped with a secondary electron (SE) detector, a backscatter electron (BSE) detector and an energy dispersive X-ray spectrometer (EDS).

(TiVZrNbHf)H_x_ was synthesized by subjecting powdered TiVZrNbHf (ball milled and sieved to <50 μm particle size) to 20 bar H_2_ at 400 °C for 48 h in an alumina crucible placed in a purpose built high pressure furnace. The chamber was flushed three times with H_2_ prior to the hydrogenation experiments. The mass of the samples were measured carefully before and after hydrogen absorption to calculate the storage capacity. The pressure-composition-isotherm (PCT) measurements were performed on the TiVZrNbHf in powder form using the SETARAM PCTPRO volumetric instrument. The absorption isotherm at 299 °C (±1) was recorded by stepwise increase of hydrogen pressure up to around 50 bar. The absorption kinetics is quite slow and the equilibrium time is set to the maximum 10 h per isotherm point. The hydrogen sorption performances were repeatable under similar pressure and temperature conditions. The thermal desorption spectroscopy (TDS) measurements have been performed by the help of a homemade instrument coupled to a quadruple mass spectrometer (MKS MicroVision Plus RGA) working under high vacuum. A constant heating ramp of 5 °C/min has been used.

Structural characterization of TiVZrNbHf and (TiVZrNbHf)H_x_ was performed with X-ray powder diffraction (XRD) with either a Bruker D8 Advance equipped with a Lynx-eye XE position sensitive detector using CuKα radiation or a Bruker D8 using a Johansson monochromator (CuKα_1_) equipped with a Lynx-eye detector. The samples for diffraction were prepared by sieving powder onto zero background single crystal silicon sample holders. The structure of the sample as-synthesized was refined from the X-ray diffraction data using the Rietveld method^9^ implemented in the FullProf^10^ program. The background was taken by linear interpolation between selected points and pseudo-Voigt peak functions were used to describe the peak shapes. For the hydrogenated samples, lattice parameters were determined using the Bruker TOPAS software. The background was refined with a 4^th^ order Chebychev polynomial and a Thompson-Cox-Hastings pseudo-Voigt^11^ peak function was used to describe the peak profile. In total 14 parameters were refined in TOPAS: 5 background coefficients, 6 peak shape parameters and the cell parameters a, b and c.

## Results

**Figure 1:**
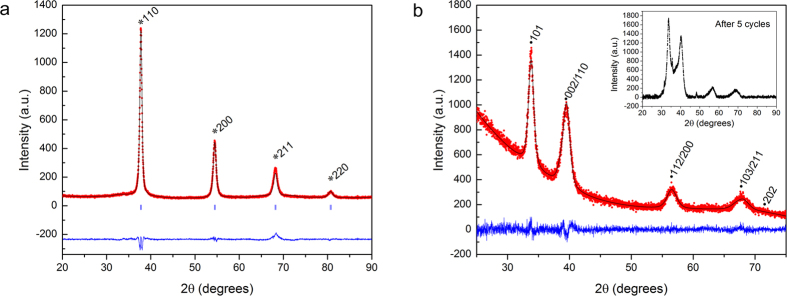
(**a**) Diffraction patterns of TiVZrNbHf as-synthesized (left), with the calculated pattern superimposed and difference curve below (λ = 1.5418 Å). (**b**), Diffraction patterns of TiVZrNbHf hydrogenated at 20 bar H_2_ and 400 °C for 48 h, peaks fitted in TOPAS (λ = 1.540598 Å), the inset shows the diffraction pattern after the fifth hydrogen absorption.

As can be seen from Fig. 1a, the XRD pattern of the alloy as-deposited indicated that TiVZrNbHf crystallizes in a simple BCC-type structure (W-type,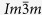) in agreement with previous studies^12^. The unit cell parameter was determined to be a = 3.3659(2) Å. No additional peaks were observed in the powder diffraction pattern showing that the sample is a single phase HEA. The chemical composition and single phase nature of the sample was analysed by SEM/EDS, the composition was confirmed to be equimolar TiVZrNbHf and EDS mapping (not shown here) verified that the material was single phase with no chemical inhomogenieties. The hydrogen storage properties of TiVZrNbHf were studied in two independent experiments: gravimetric measurements using a high pressure furnace (difference between the hydrogenated and initial sample) and volumetric pressure-composition-isotherm at 299 °C.

In the gravimetric measurements made using the high pressure furnace, the TiVZrNbHf sample absorbed hydrogen easily above 200 °C. This was observed as a decrease in the hydrogen pressure in the reactor. Treatment at 400 °C for 48 h yielded a completely hydrogenated sample with a BCT lattice (a = 3.2183(4) Å, c = 4.6556(16) Å) (Fig. 1b, right). Gravimetric measurements made before and after hydrogen absorption gave a ratio for H/M of 2.42. At first, the sample was believed to be FCC, but some of the peaks were shifted indicating a distortion of the cubic symmetry (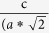 = 1.023) and thus the symmetry was lowered. All peaks could be indexed with the lower symmetry body centred tetragonal lattice (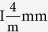). This is similar behaviour to that seen with the light rare-earth metals such as La, Ce, Pr, and Nd at H/M-ratios above 2.3 where a tetragonal distortion was noticed that can be indexed using a 1 × 1 × 2 super cell (doubling along the c-axis)^13^. A neutron diffraction study is required to completely determine the crystal structure of the hydrogenated material but this was not possible due to the large neutron absorption cross-section of Hf.

**Figure 2:**
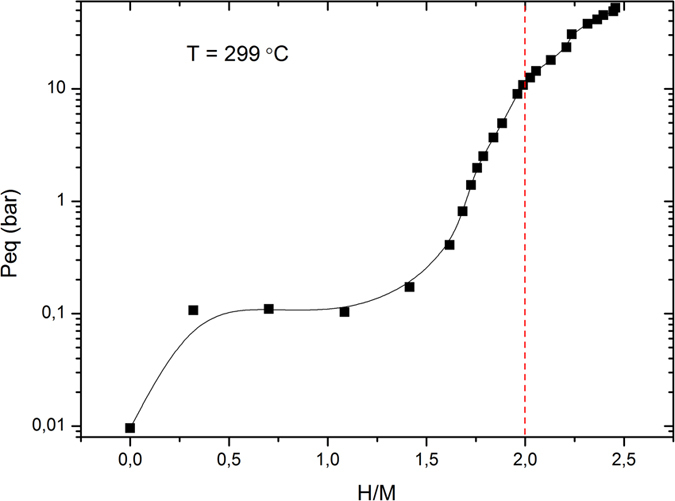
Isothermal hydrogen absorption curve for TiVZrNbHf at 299 °C. The line is added as guide to the eye.

Isothermal hydrogen absorption showed that TiVZrNbHf absorbs hydrogen readily with a plateau pressure of 0.1 bar (H_2_) at 299 °C (see Fig. 2). The plateau region extends from ~0.3–1.7 H/M. The maximum measured storage capacity was 2.5 H/M at 53 bar (H_2_), which is equivalent to 2.7 wt% hydrogen.

**Figure 3:**
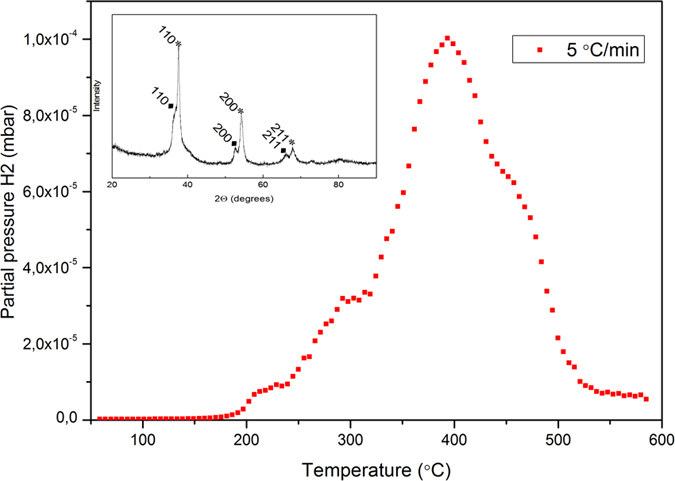
TDS spectra for hydrogen desorption from TiVZrNbHfH_x_ (red squares) with a temperature ramp rate of ~5 °C/min. The inset shows the XRD pattern of the TiVZrNbHf after desorption with a BCC-type structure (λ = 1.5418 Å).

Hydrogen desorption was investigated using TDS and it shows that hydrogen readily desorbs (with a maximum in pressure) at temperatures above 400 °C (Fig. 3) when heated at 5 °C/min and the onset of hydrogen desorption occurred for temperatures as low as 200 °C. The observed hydrogen desorption appears to be a one-step reaction causing all the hydrogen to leave the HEA simultaneously. The XRD measurements of the samples after desorption show that the hydrogen absorption/desorption is reversible and that the BCC-type structure of the sample as-synthesized was regained. However, the peaks in the XRD pattern after desorption are doublets indicating a phase separation into two different BCC phases with slightly different unit cell parameters during hydrogen desorption. The XRD pattern of TiVZrNbHf after the fifth hydrogen absorption is shown in the inset of Figure 1b. A detailed investigation of the structural stability of the alloy during hydrogen absorption-desorption cycling is currently being performed but is outside the scope of this communication.

The results presented confirm our hypothesis that an HEA can have excellent hydrogen absorption properties. It is striking that the hydrogen content in this alloy is significantly higher than in any of the binary hydrides of the constituent elements^14^,^15^,^16^,^17^,^18^. These binary hydrides have a maximum H/M ratio of 2, while we observe an H/M ratio of 2.5 in the fully hydrogenated TiVZrNbHf alloy.

## Discussion

In binary hydrides with a cubic close packed (FCC) structure such as TiH_2_ and ZrH_2_, the hydrogen is placed in tetrahedral interstitial sites. The observed H/M ratio of 2.5 in the hydrogenated TiVZrNbHf alloy requires that both tetrahedral sites and about 50% of the octahedral sites are filled with hydrogen. This behaviour is unique and has never been observed before in pure transition metal hydrides. Alloys with both transition metals and rare earth (RE) metals (lanthanides and yttrium) are known to form hydrides with H/M > 2 but our results indicate that using an HEA could be a feasible and useful strategy to avoid inclusion of RE elements in hydrogen storage materials.

**Figure 4:**
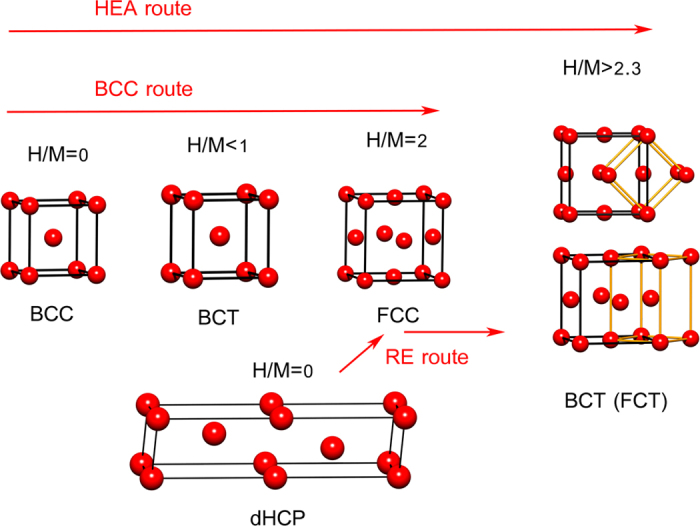
Different routes for hydrogen absorption in metals. The BCC route (V-type behaviour, BCC → distorted BCC (BCT) → FCC) up to H/M = 2. The RE route (RE = La, Ce, Pr, Nd, dHCP → FCC → distorted FCC (BTC)) with H/M>2. HEA shows a combination of the two routes (BCC → distorted FCC (BCT)).

The results above clearly show that the TiVZrNbHf alloy has a different behaviour to that of ‘normal’ transition metals. The archetypical structural transition upon creating a hydride is the formation of a FCC lattice in the fully hydrogenated form (MH_2_). However for intermediate hydrogen content, a distorted BCC phase has been observed in many transition metal-hydrogen systems (BCC → distorted BCC (BCT) → FCC)^3^,^19^,^20^. This type of lattice distortion, illustrated in Fig. 4. with an elongation of one of the cube axes has been observed in the α-β transition in the V-H system, where the c-axis is elongated by about 10%^13^. The elongation is assumed to be caused by hydrogen initially occupying octahedral sites in the BCC structure followed by a transformation to a fully hydrogenated VH_2_ phase with hydrogen in the tetrahedral sites. In the case of Ti, Zr and Hf, a tetragonal distortion of the FCC lattice (with c/a < 1) is observed for H concentrations above the critical values but with H/M < 2^13^. Wang *et al*.^21^ investigated the instability of the FCC ZrH_2_ lattice using electronic structure calculations. They found a stronger Zr-Zr bond in the FCT lattice compared to the FCC lattice that is explained by a planar type crystal field splitting in the FCT lattice and occupancy of the degenerate d_yz_ and d_xz_ orbitals.

For the light rare-earth metals such as La, Ce, Pr, and Nd, which are able to form hydrides with H/M ratio >2, another transformation route is observed (see Fig. 4). In this case, the pure metal has a double hexagonal close packed (dHCP) structure without hydrogen. Upon hydrogenation, a FCC structure is initially formed for H/M < ~2.3. At higher hydrogen contents, a tetragonal distortion is observed (dHCP → FCC → distorted FCC (BTC))^13^. This tetragonal distortion has been shown by neutron diffraction on NdD_2.36_ to result from formation of a super cell that is doubled in the c-direction^22^. In the case of CeH_2.48_ the additional hydrogen atoms were found to occupy the octahedral sites in the structure^23^.

An astonishing observation is that the TiVZrNbHf alloy based only on transition metals indicates that an HEA can exhibit a combination of these two routes. Starting with a BCC-type structure we observe a distorted FCC lattice in our fully hydrogenated HEA, where the c-axis is larger than expected (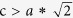, see Fig. 4), and the cube is elongated in the c-direction by ~2%, suggesting a hydrogenation path similar to the light rare-earth metals (BCC → distorted FCC (BCT)). This requires that hydrogen can be placed in both octahedral and tetrahedral sites in the HEA in contrast to other transition metal hydrides. Similar intermediate structures as in the normal BCC-case (with initial distortion of the BCC lattice) has been observed during *in situ* hydrogenations and these results are currently being evaluated, the details of this is however outside the scope of this communication. We suggest that this is due to the presence of strain in the lattice due to variations in atomic radii.

An important parameter in prediction of HEA formation is the parameter δ defined as:


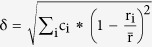


where c_i_ are the atomic composition fractions and r_i_, the atomic radii, of each component. A high value of δ leads to a large lattice distortion and makes the HEA formation less favourable. Yang and Zhang^24^ have proposed that HEAs are formed when δ < 6.6% For TiVZrNbHf δ is 6.8% (atomic radius from^25^), which is slightly above the maximum value defined by Yang and Zhang. Hence, TiVZrNbHf is expected to exhibit a highly strained lattice. Xin *et al*. have observed hydrogen occupancy in previously unavailable sites in a vanadium thin film under biaxial compressive strain. Even at low hydrogen concentrations, a change from tetrahedral to octahedral occupancy is observed^4^. The built in strain in an HEA could be the driving force to open up new interstitial sites for hydrogen. This is in agreement with previous results on, for example, the Y-H system where a change from hexagonal to cubic crystal structure is observed at very high pressures (~77 kbar)^26^. Theoretical modelling using ab initio methods is needed to explain the influence of strain on the stability of hydride formation in these types of alloys.

In summary, we have studied the hydrogenation of the high entropy alloy TiVZrNbHf and observed that extremely large amounts of hydrogen can be absorbed. The observed maximum H/M ratio of 2.5 is similar to that observed in alloys based on rare-earth metals. The formation of a distorted FCC or BCT structure in the fully hydrogenated alloy is also similar to the structure formed in rare-earth compounds. It is suggested that the unprecedented hydrogen storage capacity is an effect of the strain in the distorted HEA lattice, which favours hydrogen occupying both tetrahedral and octahedral sites. The observed H/M ratio of 2.5 corresponds only to about 2.7 wt% H. This is due to the high mass of Hf and Zr. It is likely that much higher storage capacity by weight percent can be achieved with other HEAs by replacing these elements to appropriate lighter ones. Hence, we propose that HEAs can be used as a new class of alloy for hydrogen storage that does not involve any rare-earth metals.

## Additional Information

**How to cite this article**: Sahlberg, M. *et al*. Superior hydrogen storage in high entropy alloys. *Sci. Rep*. **6**, 36770; doi: 10.1038/srep36770 (2016).

**Publisher’s note**: Springer Nature remains neutral with regard to jurisdictional claims in published maps and institutional affiliations.
